# Transcutaneous Pulsed Radiofrequency Treatment in Patients with Osteoarthritis of the Upper Extremity

**DOI:** 10.5812/aapm-146816

**Published:** 2024-08-31

**Authors:** Pleun Janssens, Iris Koenraadt-van Oost, Leonieke van Boekel, Denise Eygendaal, Annechien Beumer, Miriam van der Velden, Bertram The

**Affiliations:** 1 Department of Orthopedic Surgery, Amphia Hospital, Breda, Netherlands; 2 Foundation for Orthopedic Research, Care & Education, Amphia Hospital, Breda, Netherlands; 3 Department of Orthopedic Surgery and Sports Medicine, Erasmus Medical Centre, Rotterdam, Netherlands; 4 Amsterdam University Medical Center, Public and Occupational Health, Amsterdam, Netherlands; 5 Department of Anesthesiology, Amphia Hospital, Breda, Netherlands

**Keywords:** Transcutaneous Pulsed Radiofrequency, Osteoarthritis, Musculoskeletal Disorders, Pain

## Abstract

**Background:**

Non-invasive treatment options are preferred for managing upper extremity pain due to osteoarthritis (OA). Transcutaneous pulsed radiofrequency (TcPRF) is a promising technique and appears effective in managing knee and shoulder pain.

**Objectives:**

To investigate whether TcPRF treatment is effective in reducing pain and safe to use among patients with OA of the upper extremity.

**Methods:**

In this retrospective study, patients with painful OA of the upper extremity who underwent TcPRF treatment from February 2021 to February 2022 were included. The primary outcome measure was the change in NRS pain scores between baseline and 1, 2, and 6 weeks of follow-up. Secondary outcome measures included adverse events. Data were extracted from electronic medical records and via telephone consultation after the 6-week follow-up.

**Results:**

A total of 41 initial TcPRF treatments were performed among 37 patients. The NRS score at rest showed a statistically significant improvement at 6 weeks [median = 5 [interquartile range (IQR) 2 - 8)] compared with baseline [median = 6 (IQR 4 - 8)], P = 0.023, with a moderate effect size, r = -0.44. For NRS scores during activity, all follow-up moments had lower NRS scores [median = 7 (IQR 5 - 8)] than before TcPRF [median = 8 (IQR 7 - 9)], P = 0.002 - 0.006, with moderate to large effect sizes, r = -0.45 to r = -0.51. No adverse events were reported.

**Conclusions:**

Transcutaneous pulsed radiofrequency treatment is effective in reducing pain and is safe to use among patients with upper extremity pain due to OA.

## 1. Background

Osteoarthritis (OA) is the most common musculoskeletal disorder (1-3), affecting approximately 344 million people worldwide. Since 1990, the number of OA cases has increased by 114% (4). Osteoarthritis has significant impacts on health outcomes, including quality of life and perceived health status (1-5). Patients with OA live roughly 19 years with some form of disability (3, 6), with pain being the primary symptom (4). The prevalence of upper extremity pain is particularly high in the working population (7-9), often leading to sick leave and resulting in a socioeconomic impact on the community. In addition to work-related problems, people with upper extremity pain experience limitations in daily activities, such as dressing and carrying bags (10-14).

Given the socioeconomic impact of pain and resulting functional limitations, effective treatment is essential. Various treatment options are available, both invasive and non-invasive, with the primary aim of managing and reducing pain and functional limitations. Non-invasive treatment options are preferred due to their relative safety and low risk of complications. However, existing non-invasive treatments can sometimes be insufficient, causing patients to continue experiencing pain. Consequently, healthcare providers may be compelled to resort to invasive treatments, such as injections or surgery. Invasive treatments are less desirable, especially among the working population with upper extremity pain due to OA (7-9), as they have a higher morbidity rate, a risk of complications, and may not always result in reduced symptoms. For instance, arthroplasty for OA of the upper extremity has low implant survival rates (15). Therefore, it is necessary to search for alternative non-invasive treatment options to reduce pain and minimize discomfort for these patients.

In 2004, Balogh (16) studied a new, non-invasive method for treating pain using the transcutaneous application of pulsed radiofrequency. The proposed mechanisms of action of transcutaneous pulsed radiofrequency (TcPRF) treatment involve electric and magnetic fields (17). The electric field is most likely responsible for the effects of TcPRF, producing forces on ions and other charged structures, leading to the movement of ions and stress on cellular substructures and membranes. This movement causes ionic friction and heat, which raises tissue temperature (17, 18). Balogh's study concluded that TcPRF treatment is a promising technique and may be an option for treating patients with therapy-resistant pain (16).

In subsequent years, additional studies have shown TcPRF treatment to be effective in managing knee and shoulder pain (17-22). However, the majority of these studies did not specify the underlying diagnosis of the pain described, making it difficult to determine whether TcPRF treatment is effective for specific diagnoses.

## 2. Objectives

Therefore, the objective of this study is to investigate whether TcPRF treatment is effective in reducing pain and whether it is safe for use among patients with OA of the upper extremity.

## 3. Methods

### 3.1. Patients and Study Design

**Figure 1.**
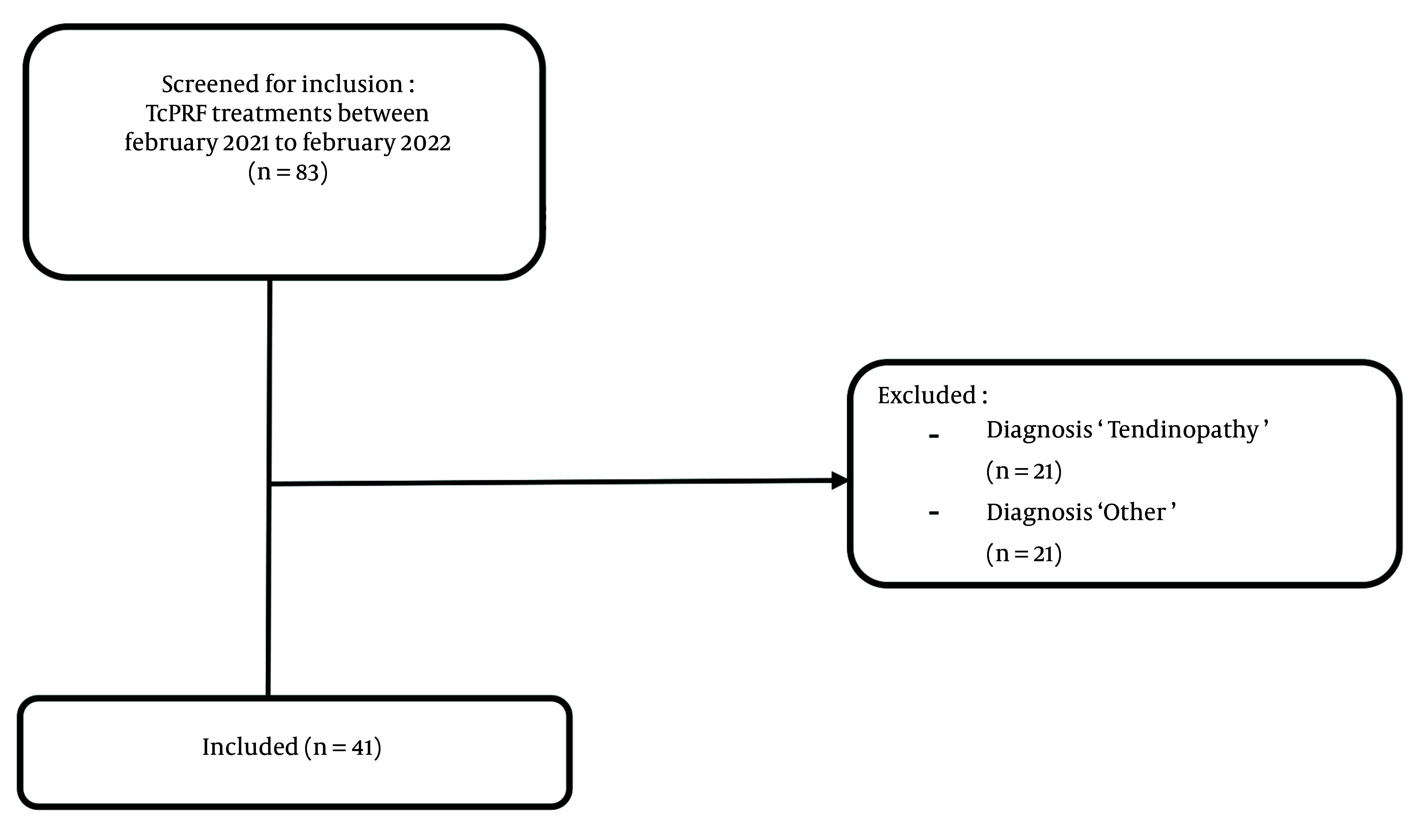
Selection process of retrospective chart review

In this single-blinded retrospective study, patients with painful OA of the upper extremity who received TcPRF treatment from February 2021 to February 2022 at the Amphia Hospital in the Netherlands were included (Figure 1). Patients diagnosed with OA of the upper extremity and persistent pain complaints were referred by the orthopedic surgeon to the pain clinic for TcPRF treatment. Before visiting the pain clinic, patients received explanatory information about the TcPRF treatment.

### 3.2. Procedure

**Figure 2.**
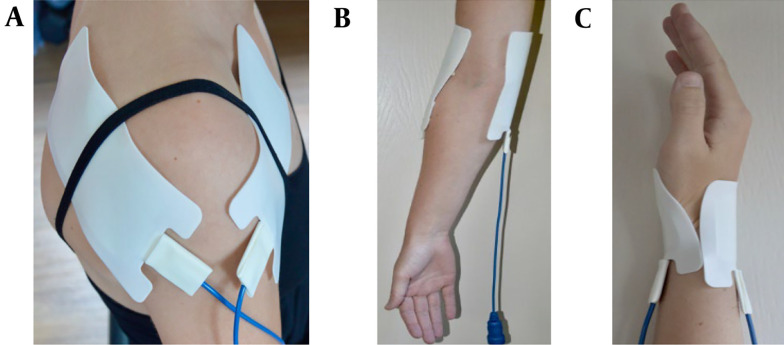
Electrode placement. A, shoulder: One medium electrode on the anterior side and one medium electrode on the posterior side of the shoulder; B, elbow: A medium electrode on the medial and lateral of the elbow joint; C, hand/wrist: A small electrode on the anterior and posterior side of the wrist

Two electrodes were attached to either the shoulder, elbow, hand, or wrist (Figure 2). The electrodes varied in size depending on the treatment location (Small: 5.5 x 5.5 cm; medium: 6.0 x 12.0 cm). These electrodes were connected to a custom-made device (Springlife medical, Utrecht, the Netherlands). Patients received a single treatment where a current of 1.4 Amperes was applied for 15 minutes [duty cycle: 15 ms (3 x 5ms/s)]. Pain management nurses performed the treatments in the pain clinic.

### 3.3. Outcome Measures

The primary outcome measure was the change in pain score, assessed using the Numeric Rating Scale (NRS) during rest and activity at baseline (T0), 1 week (T1), 2 weeks (T2), and 6 weeks (T3) of follow-up. The NRS is an 11-point scale ranging from 0, indicating no pain, to 10, indicating the worst pain imaginable. After treatment, patients received a standardized form to record their NRS scores at baseline and T1-3. Pain management nurses recorded follow-up pain scores during telephone consultations.

Secondary outcome measures, extracted from electronic medical files, included the global perceived effect (GPE), adverse events and side effects, progression to other treatment or retreatment, and satisfaction with treatment. Global perceived effect was assessed using a seven-point Likert Scale in response to the question: "To what extent have you recovered from your complaints since the start of the treatment?" with the options: Very good, good, fairly good, same as before, fairly bad, bad, and very bad.

Finally, demographic characteristics (e.g., sex, age) and clinical outcome measures were extracted from electronic medical files.

### 3.4. Medical Ethics Committee Approval

Designed as a retrospective study, formal approval from an ethical committee was not required. Additionally, the included patients did not actively object to the use of their medical files for scientific research through the opt-out function in the electronic medical record system. This manuscript adheres to the applicable STROBE guidelines.

### 3.5. Data Collection and Analyses

Descriptive analyses were used to depict the patient's characteristics and clinical characteristics at baseline. The NRS scores were tested for normal distribution using the Shapiro-Wilk test. Differences in pain between baseline and follow-up moments were investigated using paired-samples *t*-tests. Additionally, the non-parametric Wilcoxon signed-rank test was employed to examine the differences in pain between baseline and follow-up moments separately. Corresponding effect sizes were calculated for both the paired-samples t-tests and the Wilcoxon signed-rank tests. According to Cohen (23, 24), an effect size of 0.1 to 0.3 is considered a “small” effect, 0.3 to 0.5 a “moderate” effect, and > 0.5 a “large” effect. Finally, secondary outcome measures, including GPE, adverse events/side effects, progression to other treatments or retreatment, and satisfaction with treatment, were analyzed using descriptive statistics. P-values < 0.05 were considered significant. All statistical analyses were performed using the SPSS statistical package (version 25, IBM Corp, Armonk, NY, USA).

## 4. Results

In total, 37 patients were included in the study, and there were no missing data. The mean age of these patients was 53.97 ± 14.30 years (range 20 - 78). Of the included patients, 20 were female (54.1%), and 17 were male (45.9%). A total of 33 patients received unilateral treatment of one joint, 3 patients with hand/wrist pain had bilateral treatment, and 1 patient received unilateral treatment of both the elbow and hand/wrist, resulting in 41 initial TcPRF treatments. At the time of TcPRF treatment, 75.7% of the included patients had been experiencing pain for more than a year, and 12 patients (29.3%) had experienced pain for more than three years before being treated with TcPRF for the first time. Only a small percentage (14.7%) had pain complaints for less than one year. More details about the characteristics of the patients who received TcPRF treatments are displayed in Table 1. 

**Table 1.** Demographic and Clinical Characteristics of the Patients who Received Transcutaneous Pulsed Radiofrequency Treatment ^a^

Characteristics	Patients Receiving TcPRF Treatment
**Gender ** ^**b**^	
Female	20 (54.1)
Male	17 (45.9)
**Age ** ^**b**^ ** (y) **	53.97±14.30
**Diagnosis ** ^**c**^	
OA	37 (90.2)
OA + tendinopathy	4 (9.8)
**Pain spot, joint ** ^**c**^	
Shoulder	8 (19.5)
Elbow	15 (36.6)
Hand/wrist	18 (43.9)
**Duration of symptoms at baseline ** ^**c**^	
Unknown	4 (9.8)
< 3 months	0 (0.0)
3 - 6 months	2 (4.9)
7 - 11 months	4 (9.8)
1 - 2 (y)	9 (22.0)
2 - 3 (y)	10 (24.4)
> 3 (y)	12 (29.3)

Abbreviations: OA, osteoarthritis; TcPRF, transcutaneous pulsed radiofrequency.

^a^ Values are expressed as mean ± SD or No. (%).

^b^ Based on the total study population (n = 37).

^c^ Based on the number of initial TcPRF treatments (n = 41).

### 4.1. Pain

The assumption for normally distributed data was violated for almost all NRS scores since Shapiro-Wilk tests were significant, and a visual check of the histograms showed skewed distributions. Only the 1- and 2-week follow-ups of the NRS scores at rest did not violate the assumption of normal distribution [W (37) = 0.951 and 0.956; P = 0.106 and 0.152]. We performed a paired-samples *t*-test for these follow-up moments and concluded that these results were comparable to the Wilcoxon signed rank test results. To simplify, we chose to report the median and Wilcoxon signed rank test results for all follow-up moments of the NRS score.

At baseline, the median pain score at rest was lower than the pain score during activity (6 vs. 8). A Wilcoxon signed rank test revealed that NRS scores at rest were significantly lower 6 weeks after the TcPRF treatment [median = 5 (IQR 2 - 8)] compared to baseline [median = 6 (IQR 4 - 8)], z = -2.27, P = 0.023, with a moderate effect size, r = -0.44. There were no significant improvements in the NRS scores at rest for the other follow-up moments compared to baseline [T1: median = 5 (IQR 2 - 6.5), z = -1.601, P = 0.109; T2: median = 4 (IQR 2.5 - 6.5), z = -1.808, P = 0.071].

**Figure 3.**
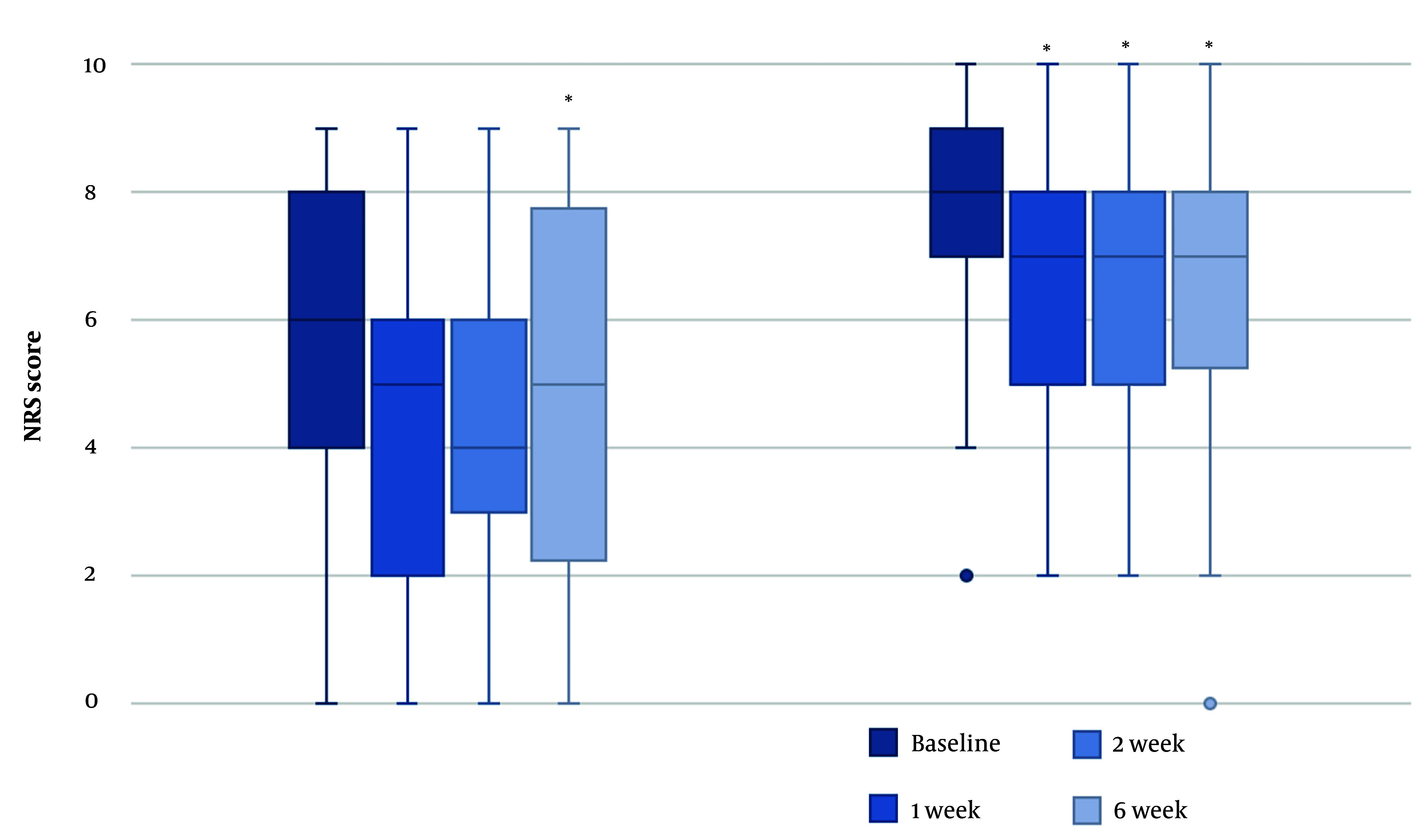
Changes in NRS score at rest (left) and during activity (right) over time. * P-value < 0.05 versus NRS score at baseline.

For NRS scores during activity, all follow-up moments had a lower NRS score [median = 7 (IQR 5 - 8)] than before TcPRF treatment [median = 8 (IQR 7 - 9)]. These differences were statistically significant (T1: z = -2.75, P = 0.006; T2: z = -2.77, P = 0.006; T3: z = -3.14, P = 0.002), with moderate to large effect sizes (T1: r = -0.45; T2: r = -0.46; T3: r = -0.51) (Figure 3). 

### 4.2. Secondary Outcome Measures

#### 4.2.1. Global Perceived Effect

Of the 37 included patients, 17 (41.5%) reported that their complaints had improved after TcPRF treatment. An equal number of patients (n = 17; 41.5%) indicated that their complaints remained the same after treatment, while 5 patients (12.2%) experienced a deterioration of their complaints. Two patients did not report their GPE (Table 2). 

**Table 2.** Global Perceived Effect ^a^

GPE	Treatments; No. (%)
**Very good**	3 (7.3)
**Good**	6 (14.6)
**Fairly good**	8 (19.5)
**Same as before**	17 (41.5)
**Fairly bad**	4 (9.8)
**Bad**	1 (2.4)
**Very bad**	0 (0.0)
**Missing**	2 (4.9)

Abbreviation: GPE, global perceived effect.

^a^ To what extent have you recovered from your complaints since the start of the treatment?

The symptom duration at baseline did not influence the GPE outcome for patients who had experienced pain for over two years. Generally, these patients reported that their complaints had either improved or stayed the same after TcPRF treatment. The GPE for patients who had experienced pain complaints for one to two years was distributed more equally, with patients being slightly more positive about their recovery than negative. We could not make any statements about whether the duration of symptoms at baseline influenced the GPE outcome for patients who had experienced pain for less than one year because the number of treatments per subgroup was too small (Appendix 1).

#### 4.2.2. Adverse Events/Side Effects

There were no adverse events reported after TcPRF treatment. In 37% of the cases, patients reported side effects during the treatment. These side effects were described as mild tingling and heat sensations around the electrodes. These side effects were only present during the treatment.

#### 4.2.3. Progression to Other Treatment or Retreatment

In 32% of the cases, there was progression to another type of treatment. Two patients received an intra-articular injection, and the others had surgical treatments such as an arthrodesis or a joint arthroplasty. Three patients received retreatment with TcPRF four months after the initial treatment.

#### 4.2.4. Satisfaction with Treatment

The majority of patients were satisfied with the TcPRF treatment. Only 9.8% of the patients were dissatisfied (Appendix 2).

## 5. Discussion

This study demonstrates that TcPRF treatment is effective in reducing pain and is a safe and viable option for patients with OA of the upper extremity. The pain score at rest showed improvement six weeks following the initial treatment, with a moderate effect size. The pain score during activity showed improvement at the one-week follow-up and persisted until the six-week follow-up, with moderate to large effect sizes. Throughout this study, no adverse events were noted, and patients were generally satisfied with the treatment.

To our knowledge, this was the first study to examine the efficacy of TcPRF treatment in patients with OA of the upper extremity. In this study, the maximum NRS difference was one point. According to a study by Salaffi et al. (25), this is considered a minimal clinically important difference (MCID) for a patient. This result is supported by the fact that patients were generally satisfied with the treatment. The majority of patients reported that their complaints had improved or remained the same after TcPRF treatment. Moreover, less than a third of the patients returned to the orthopedic surgeon to receive invasive treatment for their pain complaints.

The outcomes of changes in pain score are slightly smaller than those in previous studies on TcPRF treatment. This study found a reduction of 10% in pain scores for the NRS at rest and during activity. A previous study on shoulder pain by Taverner and Loughnan (19) found reductions of approximately 20% during activity, but did not find significant reductions in pain at rest. This difference can be explained by the fact that patients receiving TcPRF treatment for the shoulder in the study by Taverner and Loughnan (19) were treated with the electrodes positioned in six different directions. Because of this, an electric and magnetic field is created at six different angles in the shoulder joint. The proposed mechanisms of action, as mentioned before, of TcPRF treatment are the electric and magnetic fields (17). The electric field is most likely responsible for all the effects of TcPRF. The electric field produces forces on ions and other charged structures, causing movement of ions and stress on cellular substructures and membranes. The movement of ions causes ionic friction and heat, which in turn raises tissue temperature (17, 18). If treatment is performed at six different angles, more cells are involved, leading to improved outcomes. Consequently, with fewer angles, the improvement of outcomes would be lesser.

In current literature about the efficacy of TcPRF treatment, TcPRF is used as a last-resort treatment before surgical intervention (18-20). This also applied to the current study, in which the majority (75.7%) of the patients had persistent pain complaints for over one year. The orthopedic surgeons referred these patients to the pain clinic since alternative non-invasive treatment methods had failed to produce satisfactory results. Taverner et al. (18) suggested using TcPRF as an early option in the course of treatment in combination with physiotherapy when pain complaints are delaying rehabilitation without compromising other treatment options. Since TcPRF is a non-invasive treatment with no major adverse events, it should be considered an earlier treatment option for OA.

The findings of this study should be interpreted while considering several possible limitations. Firstly, the retrospective study design and small population limit the generalizability of the results. However, TcPRF treatment for patients with painful OA of the upper extremity was only introduced at the beginning of 2021. Secondly, recall bias could have influenced the patients’ reports of their NRS scores. Patients were not required to submit their pain scores until the six-week follow-up. If they had not written down their pain scores at the follow-up moment, these scores might have been influenced by how they felt during the telephone consultation. Nonetheless, patients were extensively informed at the pain clinic about the follow-up period. In addition, the follow-up period was relatively short, making the chance of recall bias acceptable. Despite these limitations, this study, which used the minimal dosage of TcPRF, found the MCID for patients and thus provides an understanding of the efficacy of TcPRF treatment.

Future research is necessary to further understand the benefits and effects of this treatment. Firstly, it should focus on how the procedure should be performed (e.g., dosage, treatment frequency and time, electrode placements). Secondly, future research is necessary to study the optimal timing of TcPRF treatment in the course of OA. Lastly, insights into the effect of TcPRF treatment on objective outcome measures, such as range of motion, are needed.

### 5.1. Conclusions

In conclusion, TcPRF treatment seems to be a beneficial addition to the options for treating OA of the upper extremity. It reduces pain and is a safe treatment option for patients with pain complaints of the upper extremity due to OA.

aapm-14-4-146816-s001.pdf

## Data Availability

The dataset presented in the study is available on request from the corresponding author during submission or after publication.

## References

[1] Martel-Pelletier J, Barr AJ, Cicuttini FM, Conaghan PG, Cooper C, Goldring MB (2016). Osteoarthritis.. Nat Rev Dis Primers..

[2] Pereira D, Ramos E, Branco J (2015). Osteoarthritis.. Acta Med Port..

[3] Australian Institute of Health and Welfare (2021). Osteoarthritis Citation AIHW ..

[4] Cieza A, Causey K, Kamenov K, Hanson SW, Chatterji S, Vos T (2021). Global estimates of the need for rehabilitation based on the Global Burden of Disease study 2019: a systematic analysis for the Global Burden of Disease Study 2019.. Lancet..

[5] Altman RD (2010). Early management of osteoarthritis.. Am J Manag Care..

[6] Arden N, Nevitt MC (2006). Osteoarthritis: epidemiology.. Best Pract Res Clin Rheumatol..

[7] Bot SD, van der Waal JM, Terwee CB, van der Windt DA, Schellevis FG, Bouter LM (2005). Incidence and prevalence of complaints of the neck and upper extremity in general practice.. Ann Rheum Dis..

[8] Greving K, Dorrestijn O, Winters JC, Groenhof F, van der Meer K, Stevens M (2012). Incidence, prevalence, and consultation rates of shoulder complaints in general practice.. Scand J Rheumatol..

[9] Engebretsen KB, Grotle M, Natvig B (2015). Patterns of shoulder pain during a 14-year follow-up: results from a longitudinal population study in Norway.. Shoulder Elbow..

[10] Picavet HS, Schouten JS (2003). Musculoskeletal pain in the Netherlands: prevalences, consequences and risk groups, the DMC(3)-study.. Pain..

[11] Martimo KP, Shiri R, Miranda H, Ketola R, Varonen H, Viikari-Juntura E (2009). Self-reported productivity loss among workers with upper extremity disorders.. Scand J Work Environ Health..

[12] Feleus A, Miedema HS, Bierma-Zeinstra SM, Hoekstra T, Koes BW, Burdorf A (2017). Sick leave in workers with arm, neck and/or shoulder complaints; defining occurrence and discriminative trajectories over a 2-year time period.. Occup Environ Med..

[13] Baldwin ML, Butler RJ (2006). Upper extremity disorders in the workplace: costs and outcomes beyond the first return to work.. J Occup Rehabil..

[14] Walker-Bone K, Palmer KT, Reading I, Coggon D, Cooper C (2004). Prevalence and impact of musculoskeletal disorders of the upper limb in the general population.. Arthritis Rheum..

[15] Interventies LRO (2021). Annual report 2021..

[16] Balogh SE (2004). Transcutaneous application of pulsed radiofrequency: four case reports.. Pain Pract..

[17] Cosman ER, Cosman ER (2005). Electric and thermal field effects in tissue around radiofrequency electrodes.. Pain Med..

[18] Taverner MG, Loughnan TE, Soon CW (2013). Transcutaneous application of pulsed radiofrequency treatment for shoulder pain.. Pain Pract..

[19] Taverner M, Loughnan T (2014). Transcutaneous pulsed radiofrequency treatment for patients with shoulder pain booked for surgery: a double-blind, randomized controlled trial.. Pain Pract..

[20] Taverner MG, Ward TL, Loughnan TE (2010). Transcutaneous pulsed radiofrequency treatment in patients with painful knee awaiting total knee joint replacement.. Clin J Pain..

[21] Liu A, Zhang W, Sun M, Ma C, Yan S (2016). Evidence-based Status of Pulsed Radiofrequency Treatment for Patients with Shoulder Pain: A Systematic Review of Randomized Controlled Trials.. Pain Pract..

[22] Lin ML, Chiu HW, Shih ZM, Lee PY, Li PZ, Guo CH (2019). Two Transcutaneous Stimulation Techniques in Shoulder Pain: Transcutaneous Pulsed Radiofrequency (TPRF) versus Transcutaneous Electrical Nerve Stimulation (TENS): A Comparative Pilot Study.. Pain Res Manag..

[23] Cohen J (1992). A power primer.. Psychol Bull..

[24] Cohen J (1988). Statistical power analysis for the behavioral sciences..

[25] Salaffi F, Stancati A, Silvestri CA, Ciapetti A, Grassi W (2004). Minimal clinically important changes in chronic musculoskeletal pain intensity measured on a numerical rating scale.. Eur J Pain..

