# Supplementary material for: Transcutaneous Pulsed Radiofrequency Treatment in Patients with Osteoarthritis of the Upper Extremity
Source: Anesth Pain Med. 2024 Aug 31;14(4):e146816. doi: 10.5812/aapm-146816 (PMC11895798; doi:10.5812/aapm-146816)
Supplement: aapm-14-4-146816-s001.pdf [file aapm-14-4-146816-s001.pdf]

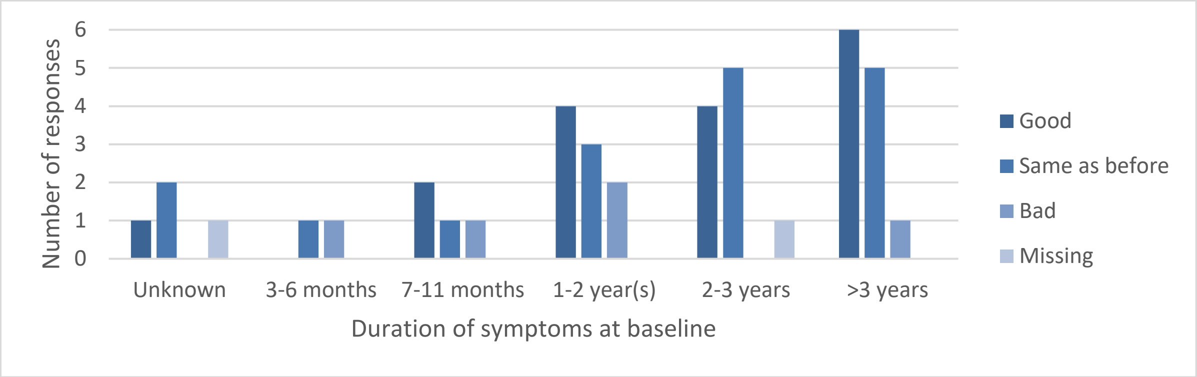

Appendix 1. The number of responses for each GPE outcome for the duration of symptoms at baseline

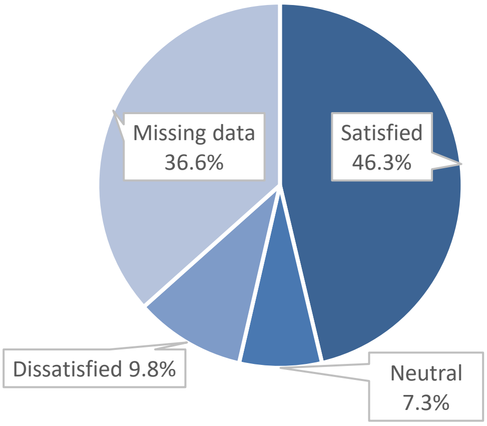

Appendix 2. Satisfaction with treatment
